# T54R mutation destabilizes the dimer of superoxide dismutase 1^T54R^ by inducing steric clashes at the dimer interface

**DOI:** 10.1039/c9ra09870d

**Published:** 2020-03-13

**Authors:** Debasish Kumar Ghosh, Abhishek Kumar, Akash Ranjan

**Affiliations:** Computational and Functional Genomics Group, Centre for DNA Fingerprinting and Diagnostics Uppal Hyderabad 500039 Telangana India akash@cdfd.org.in +91-40-27216006 +91-40-27216159; Graduate Studies, Manipal Academy of Higher Education Manipal Karnataka 576104 India

## Abstract

Mutations cause abnormalities in protein structure, function and oligomerization. Different mutations in the superoxide dismutase 1 (SOD1) protein cause its misfolding, loss of dimerization and aggravate its aggregation in the amyotrophic lateral sclerosis disease. In this study, we report the mechanistic details of how a threonine-to-arginine mutation at the 54^th^ position (T54R) of SOD1 results in destabilization of the dimer interface of SOD1^T54R^. Using computational and experimental methods, we show that the T54R mutation increases fluctuation of the mutation-harboring loop (R54-loop) of SOD1^T54R^. Fluctuation of this loop causes steric clashes that involve arginine-54 (R54) and other residues of SOD1^T54R^, resulting in loss of inter-subunit contacts at the dimer interface. Since the T54 residue-containing loop is necessary for the dimerization of wild-type SOD1, fluctuation of the R54-loop, steric clashes involving R54 and loss of inter-subunit contacts give rise to the loss of SOD1^T54R^ dimer stability. This correlates to energetically unfavorable tethering of the monomers of SOD1^T54R^. The outcome is gradual splitting of SOD1^T54R^ dimers into monomers, thereby exposing the previously buried hydrophobic interface residues to the aqueous environment. This event finally leads to aggregation of SOD1^T54R^. T54R mutation has no effect in altering the relative positions of copper and zinc ion binding residues of SOD1^T54R^. The native SOD1 structure is stable, and there is no destabilizing effect at its dimer interface. Overall, our study reveals the intricate mechanism of T54R mutation-associated destabilization of the dimer of the SOD1^T54R^ protein.

## Abstract 2

T54R mutation destabilizes the dimer of SOD1^T54R^.

## Graphical abstract

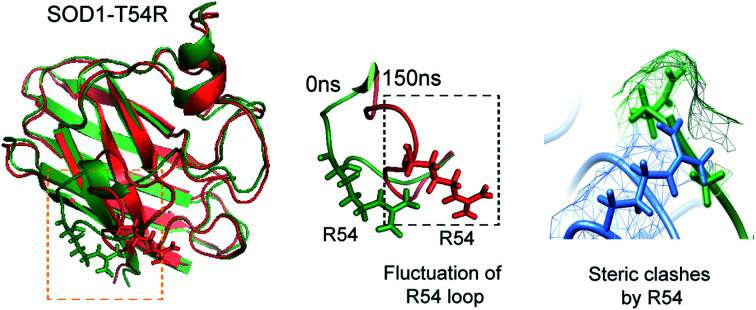

## Introduction

The hierarchy of the stages of proper protein folding not only encompasses the correctness of secondary structures,^1^ but it also accounts for the stabilization of tertiary and oligomeric structures of proteins.^2^ While the stability of a protein's secondary structure is governed by its intrinsic sequence feature, the accuracy of tertiary and quaternary structures is regulated by molecular and environmental factors.^3^ The order of a protein's folding and dynamic stability also depends upon complex cellular machineries like chaperones and post-translational modifiers.^4,5^ The events of regulated and stoichiometrically defined oligomerization of proteins are required for their optimal stability and functionality.^6^ In a stable oligomer, critical residues at the interface of subunits mediate necessary interactions with the residues of other subunits. Precise interactions of interface-residues determine the spatiotemporal quality of protein oligomers *in vitro* and *in vivo*. Fluctuation of environmental factors and mutations in protein sequences generate detrimental effects upon the conformation and stability of protein oligomers.^7,8^ Harmful mutations in proteins can collapse their structures,^9^ resulting in loss of oligomeric associations. Dissociation of oligomers of mutant protein is often a result of destabilization of the oligomerization interface.^10^ The instability of oligomerization interface is coupled to different mechanisms like steric clashes of the interface residues, loss of inter-molecular interactions, alteration of secondary structures at interface *etc.*^11^ In case of some proteins, dissociated monomers are prone to undergo aggregation in physiological condition.^12^ Dissociation of oligomers into monomers induces an aberrant exposure of hydrophobic regions of monomers to aqueous environment.^13^ Such hydrophobic regions can be aggregation-prone, leading to aggregation of the proteins.^14^ In this study, we have characterized the effects of threonine-to-arginine mutation at the 54^th^ position (T54R) of superoxide dismutase 1 (SOD1^T54R^) upon the dimer stability of the protein, other than finding the mechanism of induction of aggregation properties in SOD1^T54R^.

Superoxide dismutase 1 (SOD1) protein scavenges the radical superoxide ion and reduces intracellular oxidative stress.^15^ SOD1 binds to divalent copper and zinc ions to catalyze the conversion of superoxide ion to molecular oxygen.^16^ SOD1 has an eight-stranded beta-barrel structure with three catalytically active regions – copper ion binding residues, zinc ion binding residues and substrate-guiding electrostatic loop. SOD1 is a homodimeric protein. Dimerization and maturation of SOD1 is mediated by interaction of dimerization-promoting residues of the monomers, metalation and specific inter-subunit disulfide linkage.^17^ There are reports of different mutations in SOD1 sequence.^18^ Many of the mutations of SOD1 are known to destabilize its structure and transform the protein into an aggregation-prone entity. Aggregation of mutant SOD1 protein in the motor neuron cells is linked to the initiation and progression of amyotrophic lateral sclerosis (ALS) disease.^19^ Toxic mutations are observed to be dispersed throughout the SOD1 sequence, and they accelerate aggregation of the protein by different mechanisms.^20^ For example, mutations cause local structural unfolding,^21^ aberrant exposure of hydrophobic segments to aqueous environment,^22^*etc.* which lead to prion-like aggregation of mutant SOD1 proteins.^23^ While many studies on mutant SOD1 describe the impact of the protein's aggregation in ALS, a few have also shown how the misfolded monomeric intermediates of mutant SOD1 are responsible for the protein's aggregation.^24,25^ However, the mechanism of dimer-to-monomer transition of mutant SOD1 proteins is not completely understood. Mutations which reside outside of the dimer interface can induce conformational alterations in SOD1 structure, leading to destabilization of the dimer.^26^ Though the effects of such mutations, like G85R, G93A *etc.* upon SOD1 stability and aggregation are vividly studied,^27^ the effects of mutations at dimer interface of SOD1 are not thoroughly explored. Since dimerization of SOD1 has significant implication in its catalytic activity and aggregation prevention,^28^ it is necessary to understand the effects of interface-residing mutations, such as T54R, upon the stability of SOD1.

In this study, we have investigated how the T54R mutation manifests weakening effects upon the stability of SOD1^T54R^ dimer. T54R mutation destabilizes the dimer interface due to the reason that arginine-54 (R54) is involved in multiple steric clashes with the nearby residues. The steric clashes subsequently trigger the loss of intra- and inter-subunit interactions, resulting in increased dimer-to-monomer transition of SOD1^T54R^. Several hydrophobic residues in the unstable SOD1^T54R^ dimer are exposed to aqueous environment, and they cause aberrant aggregation of the protein.

## Results

### Fluctuation of the T54R mutation-containing loop results in destabilization of the dimer interface of SOD1^T54R^

Many of the ALS-associated mutations of SOD1, like G85R and G93A, enhance aggregation of the protein. However, the effects of some other mutations, like T54R, C57S *etc.* upon SOD1 stability are not known. In this study, we endeavored to understand the effects of T54R mutation upon the structural stability of SOD1 due to the reason that it is a naturally occurring mutation, and this mutation is also linked to SOD1^T54R^-mediated induction of ALS disease.^29^ Since the effects of this previously reported mutation at SOD1 dimer interface is not known in detail, we analyzed what effects T54R mutation has upon the monomeric and dimeric structures of SOD1^T54R^. Because molecular dynamics simulation (MDS) can be an invaluable tool to study the stability of dimeric proteins,^30,31^ we used this method and other computational as well as experimental techniques in this study.

In the wild-type SOD1 protein, threonine-54 (T54) residue is situated in an unstructured loop (T54-loop) that connects the fourth and fifth beta-sheets of the eight-stranded beta barrel structure. Residues of this loop region participate in the formation of SOD1 dimer interface by interacting with the residues of first beta-sheet of the other subunit. Hence, this loop is important for mediating inter-subunit association and dimerization of SOD1. T54R mutation changes the residue property (polar-to-charged) and geometry (smaller-to-larger side chain) of the amino acid at 54^th^ position of SOD1^T54R^ compared to SOD1. Hence, we hypothesize that T54R mutation has modulatory effects in the stability of SOD1^T54R^ dimer.

**Fig. 1:**
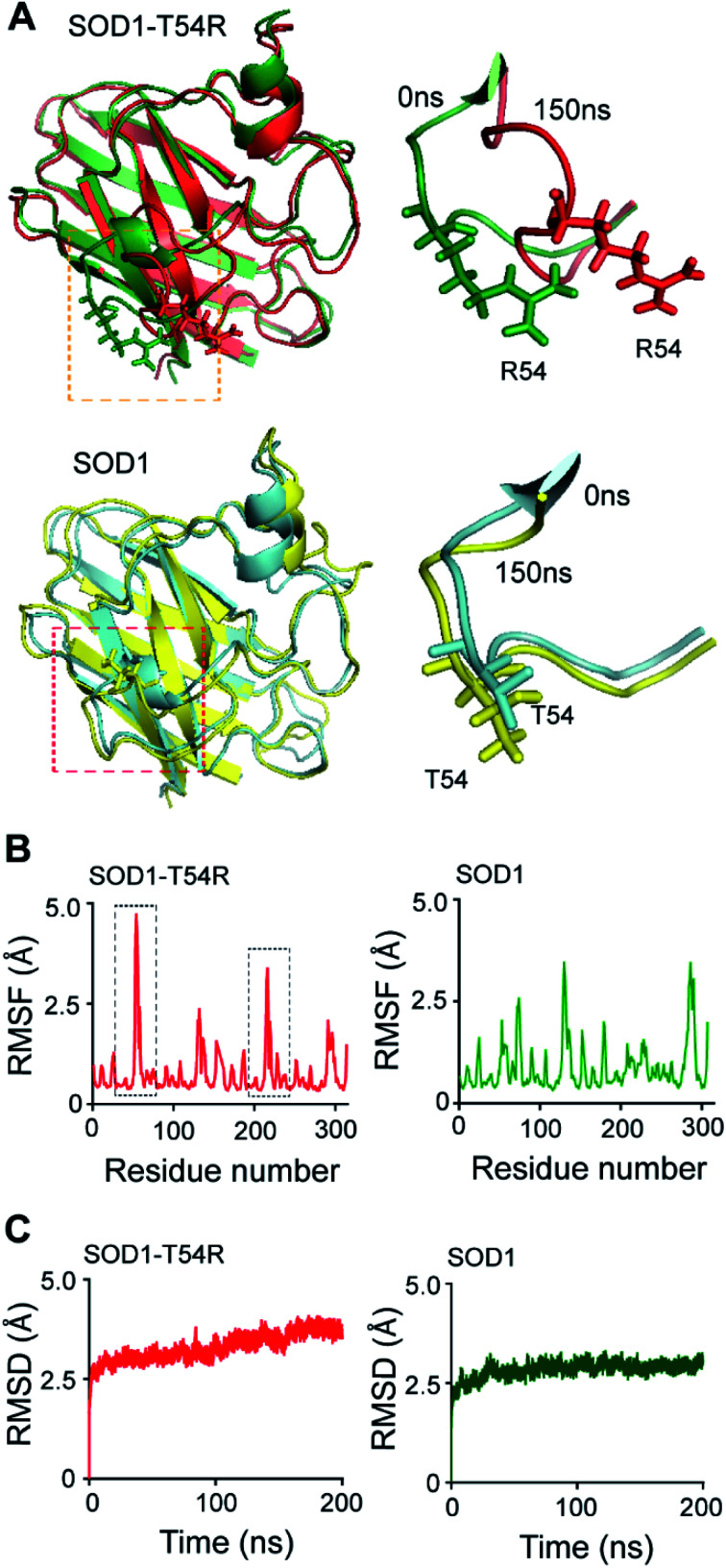
R54-loop of SOD1^T54R^ fluctuates during the molecular dynamics simulation. (A) [Upper panel] Superimposed 0 ns and 150 ns structures of SOD1^T54R^. [Lower panel] Superimposed 0 ns and 150 ns structures of SOD1. (B) Root mean square fluctuation [RMSF] of the residues of SOD1^T54R^ and SOD1 dimers during MDS [black boxes show highly fluctuating residues of SOD1^T54R^]. (C) Root mean square deviation [RMSD] of the backbones of SOD1^T54R^ and SOD1 dimers during simulation.

In the MDS studies, we observed that the loop harboring R54 of SOD1^T54R^ (R54-loop) showed time-dependent fluctuation from its original position (Fig. 1A). At several time points of the later stages of simulation, the position of R54-loop deviated from its initial/starting location. Deviation of R54 of the two subunits of SOD1^T54R^ dimer was not identical. While R54 of one subunit (subunit-1) fluctuated as much as 4.72 Å from its initial position, R54 of the other subunit (subunit-2) showed a maximum fluctuation of 3.06 Å from its initial position (Fig. 1B). It was noted that the neighboring residues of R54 had also undergone high fluctuation which was represented by higher root mean square fluctuation (RMSF) values of the residues of R54-loop region (Fig. 1B). The 52^nd^–59^th^ residues of subunit-1 and subunit-2 showed RMSF values ranging in between 1.16–4.72 Å and 0.9–3.37 Å respectively. Since this region was unstructured, it was highly dynamic in terms of acquiring different positions at different time points of simulation. T54R mutation had increased the fluctuation of this region even more by participating in additional favorable and unfavorable interactions with the nearby residues. Depending upon differential interactions with the nearby water molecules and other residues of the subunits, the cumulative effect of stabilizing and destabilizing interactions of the residues of R54-loop region could be different in the two subunits of SOD1^T54R^ dimer. This had resulted in high, but nonidentical, fluctuation of R54-loop regions in the two monomers of SOD1^T54R^ dimer. We observed that SOD1^T54R^ dimer became stable only in the later phase (after 150 ns) of simulation. This was manifested by initial increase followed by maintenance of maximum root mean square deviation (RMSD) values of the backbone of SOD1^T54R^ dimer (Fig. 1B). We understood that the initial instability of SOD1^T54R^ dimer was not associated with structural deformation of individual monomers, but because of the increased fluctuation of R54-loop. This was evident from the fact that the beta barrel of each subunit of SOD1^T54R^ dimer was intact even in the simulation phase that showed instability of SOD1^T54R^ dimer backbone and high fluctuation of R54-loop (Fig. 1A).

The wild-type SOD1 dimer was stable throughout the simulation time. At different time points of simulation, the overall structure of SOD1, including the T54-loop, did not show high fluctuation (Fig. 1A and B). RMSF values of the residues of subunit-1 and subunit-2 were in the range of 1.1–3.45 Å and 1.35–3.45 Å respectively. The residues of each subunit of SOD1 dimer did not show high RMSF values, except the electrostatic loop region that spanned from 127^th^–138^th^ residues (Fig. 1B). This observation was in-line with our previous finding in which we had shown that the electrostatic loop region was the most unstable region of native SOD1 protein.^21^ RMSD values of the backbone of SOD1 dimer represented a highly stable dimer (Fig. 1C). The backbone attained maximum stability within 30 ns of the simulation start time. The highest RMSD value of SOD1 dimer (2.64 Å) was also lower than the highest RMSD value of SOD1^T54R^ dimer (3.62 Å). Hence, it was evident that SOD1^T54R^ dimer was more unstable than wild-type SOD1 dimer.

**Fig. 2:**
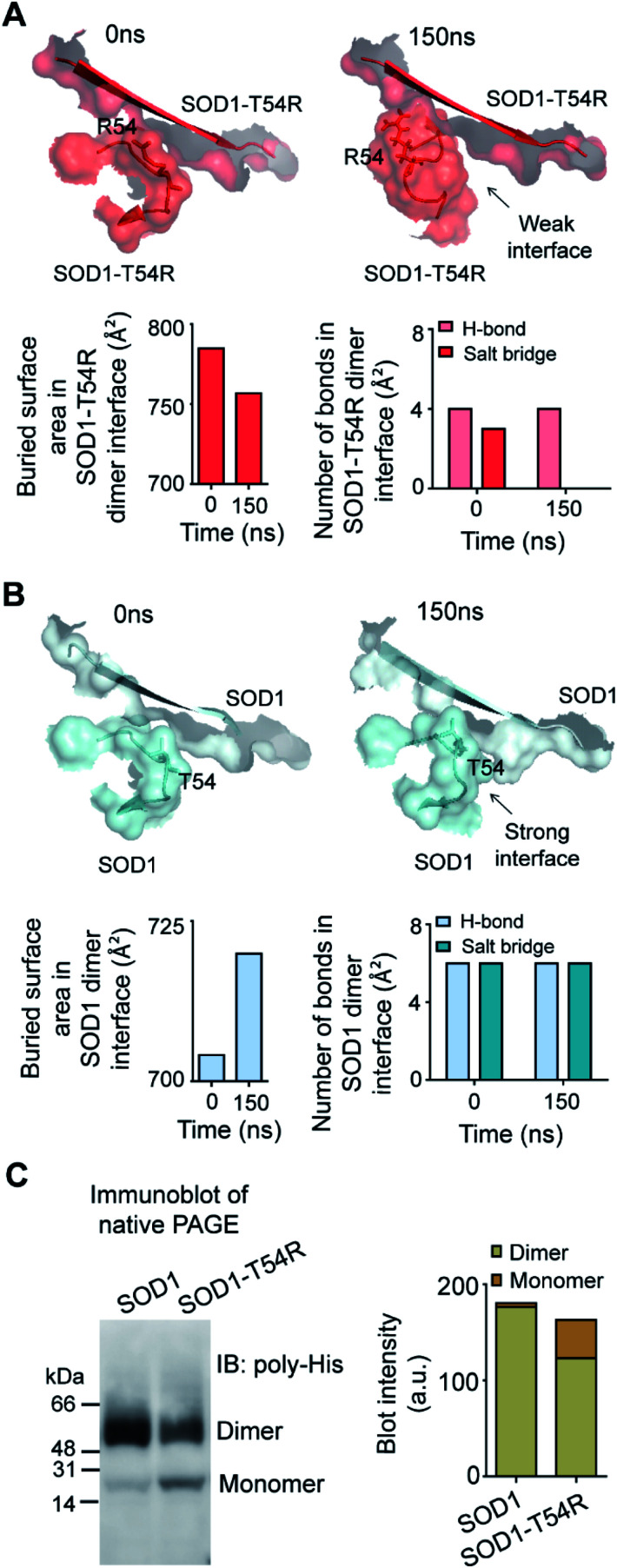
The dimer interface of SOD1^T54R^ is weakened due to displacement of R54-loop distant from dimer interface, leading to dimer-to-monomer conversion of SOD1^T54R^. (A) [Upper panel] Gradual positioning of R54-loop distant from the dimer interface of SOD1^T54R^ during the late phase of simulation. [Lower panel] Quantification of buried surface area and number of noncovalent bonds at dimer interface of SOD1^T54R^ during different time points of MDS. (B) [Upper panel] Constant positioning of T54-loop at the interface of SOD1 dimer during MDS. [Lower panel] Quantification of buried surface area and number of noncovalent bonds at dimer interface of SOD1 during different time points of simulation. (C) Immunoblot of recombinant SOD1 and SOD1^T54R^ proteins.

Fluctuation of R54-loops of the subunits of SOD1^T54R^ dimer had direct impacts on the stability of dimer interface. The fluctuation had resulted in positioning of the loop distant from the first beta-sheet of the other subunit, thereby destroying the dimer interface (Fig. 2A). Individual subunits of SOD1^T54R^ dimer were observed to be loosely tethered with each other in the R54-loop repositioned conformation (Fig. 2A). Buried surface area of the interface of SOD1^T54R^ dimer decreased with the progression of simulation (Fig. 2A). Fluctuation of R54-loop also resulted in loss of hydrogen bonds (H-bonds) and salt bridges that existed between the interface residues in the initial conformation of SOD1^T54R^ dimer (Fig. 2A). Not only the H-bonds and polar contacts but the number of total direct contacts (including hydrophobic and van der Waals interactions) between the residues of two monomers at dimer interface of SOD1^T54R^ also decreased with the progression of simulation (dimer interface contacts at: 0 ns = 11, 150 ns = 5). This implied that the dimer interface was gradually disrupted with continuous shifting of R54-loop distant from the interface. In the wild-type SOD1 dimer, T54-loop did not fluctuate drastically. T54-loop was observed to be tightly tethered to the first beta-sheet of the other subunit (Fig. 2B). Since buried surface area of the interface of SOD1 dimer increased during the simulation (Fig. 2B), it was evident that tethering of T54-loop and the first beta-sheet of the other subunit became stronger with time. The number of H-bonds and salt bridges between the subunits of SOD1 at dimer interface remained unchanged over the simulation time (Fig. 2B). The total number of direct contacts (including hydrophobic and van der Waals interactions) between the residues of the monomers of SOD1 dimer also remained constant throughout the simulation time (dimer interface contacts at: 0 ns = 16, 150 ns = 16).

We conducted experiments to validate if SOD1^T54R^ dimer had less stability that could result in its dissociation into monomers. When an equal mass (40 μg) of recombinant SOD1 and SOD1^T54R^ proteins were subjected to a native poly-acrylamide gel electrophoresis (native-PAGE) for separation of the monomers and dimers of each protein, we observed that almost entire SOD1 protein existed in dimeric form (Fig. 2C). Though a fraction of SOD1^T54R^ existed as dimer, we found that a high fraction of SOD1^T54R^ also remained as monomer (Fig. 2C). This proved that SOD1^T54R^ dimer was intrinsically more unstable than wild-type SOD1 dimer. Unstable SOD1^T54R^ dimer had undergone more dissociation into monomers. This observation concurred with the results of MDS which showed that fluctuation of R54-loop could lead to destabilization of the dimer interface, leading to dissociation of dimeric SOD1^T54R^ to monomers.

**Fig. 3:**
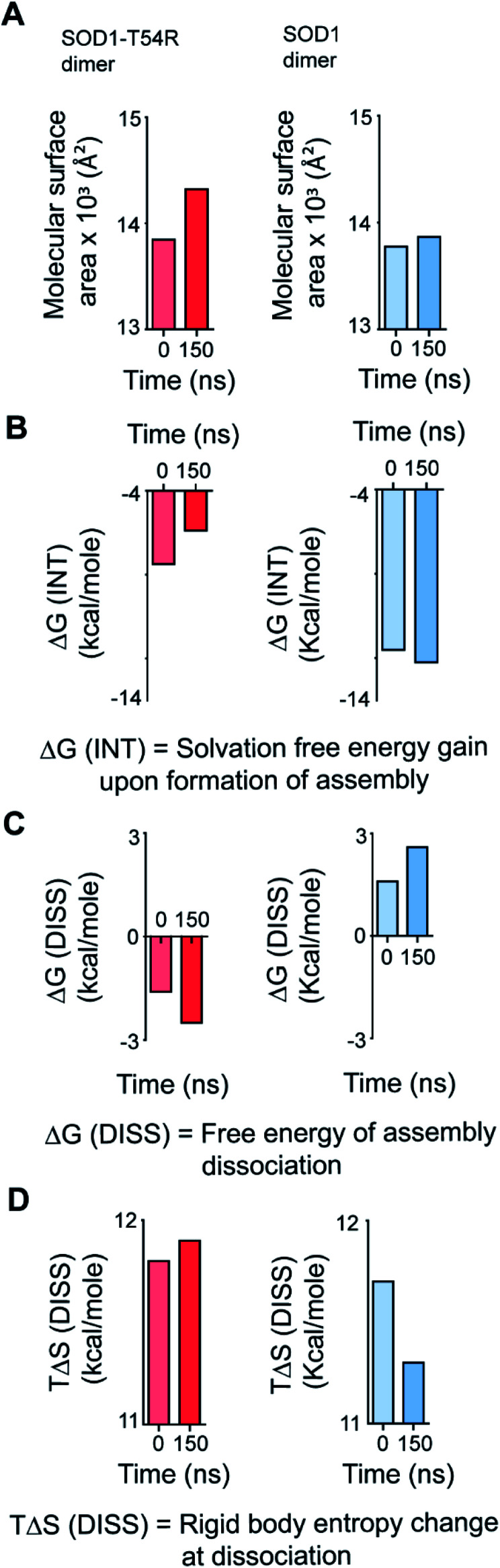
Different structural and thermodynamic parameters show that the dimer interface of SOD1^T54R^ becomes unstable with progression of MDS. The dimer interface of SOD1 becomes more stable during progression of MDS. (A) Quantification of molecular surfaces of SOD1^T54R^ and SOD1 dimers during different time points of MDS. (B) Quantification of loss of solvation free energy gain [Δ*G*^INT^] for the formation of SOD1^T54R^ and SOD1 dimer at 0 ns and 150 ns of MDS. (C) Quantification of the negative free energy of dissociation [Δ*G*^DISS^] of SOD1^T54R^ and SOD1 dimers during different simulation time points. (D) Quantification of the entropy [*T*Δ*S*] of the R54-loop of SOD1^T54R^ and T54-loop of SOD1 during different time points of simulation.

We calculated the molecular surfaces and thermodynamic parameters of SOD1^T54R^ and SOD1 dimers at different time points of MDS. It was observed that the total molecular surface area of SOD1^T54R^ dimer gradually increased in the later stages of MDS (Fig. 3A). We understood that the increase of total molecular surface area of SOD1^T54R^ dimer was due to the weakening of dimer stability and gradual exposure of dimer interface to aqueous environment. While the dimer interface of SOD1^T54R^ was inaccessible to water in the early stages of MDS, a continuous displacement of R54-loop from the interface had caused an opening of the interface, leading to increment of molecular surface of SOD1^T54R^ dimer. On the contrary, molecular surface of SOD1 did not change during the entire simulation time (Fig. 3A). Although the buried dimer interface area of SOD1 increased during simulation, we also observed that fluctuation of the electrostatic loop of each monomer had exposed some previously buried area to the aqueous environment. Overall, burial of more surface area near the dimer interface had compensated for the exposure of surface area near the electrostatic loop, thereby keeping the total solvent exposed molecular surface area of SOD1 dimer almost unaltered. Solvation free energy gain upon formation of assembly [Δ*G* (INT)] also showed the unstable nature of SOD1^T54R^ dimer interface. Δ*G* (INT) represented the solvation free energies of monomeric and dimeric forms of SOD1^T54R^. A higher negative Δ*G* (INT) value signified higher reciprocal affinity of monomers in the dimer due to hydrophobic interactions. Δ*G* (INT) values of SOD1^T54R^ increased with the progression of MDS (Fig. 3B). This showed that the energetic stability of SOD1^T54R^ dimer interface due to hydrophobic interactions was lowered. However, SOD1 dimer showed higher interface stability in the MDS. Δ*G* (INT) values of SOD1 dimer decreased during the advancement of simulation time (Fig. 3B). Free energy of dissociation [Δ*G* (DISS)] values also pointed that SOD1^T54R^ dimer was prone to dissociation (Fig. 3C). By convention, Δ*G* (DISS) < 0 represented thermodynamically unstable assemblies. While the starting structure of SOD1^T54R^ itself had a negative Δ*G* (DISS) value, the Δ*G* (DISS) of SOD1^T54R^ dimer further decreased with the progression of MDS. This implied that the intrinsically unstable SOD1^T54R^ dimer became more labile with time. The wild-type SOD1 dimer was stable in terms of its Δ*G* (DISS) values. Δ*G* (DISS) values of SOD1 during different time points of simulation showed increasingly positive values (Fig. 3C). The rigid-body entropy changes at dissociation (*T*Δ*S*) of SOD1^T54R^ dimer increased during simulation (Fig. 3D). On the contrary, *T*Δ*S* of SOD1 dimer decreased during simulation (Fig. 3D). This also gave an evidence that SOD1^T54R^ dimer was predisposed to dissociation into monomers which increased the entropy of the system. SOD1 dimer was more compact, and it was less susceptible to dissociation into monomers.

### Steric clashes between R54 and other residues cause loss of inter-subunit interactions at the dimer interface of SOD1^T54R^

We investigated how the fluctuation of R54-loop caused the instability of SOD1^T54R^ dimer. Since arginine had a longer side chain than threonine, we expected that translation and torsional movements of the side chain of R54 would allow it to contribute in a greater number of steric clashes.

**Fig. 4:**
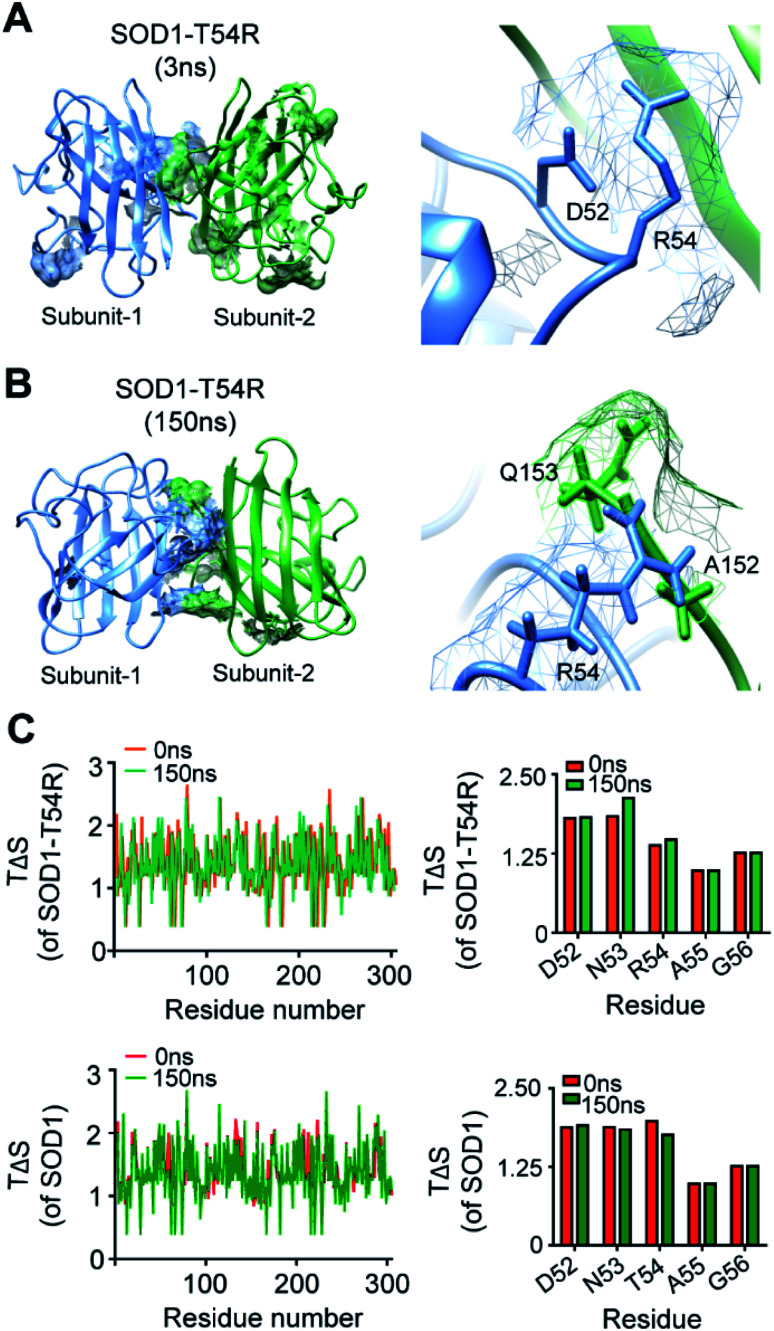
The arginine-54 residue of R54-loop is involved in steric clashes with the nearby residues. (A and B) The steric clashes of R54 of SOD1^T54R^ with different intra- and inter-subunit residues [(A) – steric clash with D52 of same subunit, (B) – steric clashes with A152 and Q153 of other subunit] at different time points of MDS. (C) Conformational entropy [*T*Δ*S*] of the residues of SOD1^T54R^ and SOD1 dimers at different time points of MDS.

We observed that R54 of SOD1^T54R^ was involved in many unfavorable intra- and inter-subunit steric collisions with its neighboring amino acids. In the early stage of simulation, R54 showed steric clashes with the nearby residues [such as Asp-52 (D52)] of same subunit (Fig. 4A). After the repositioning of R54-loop distant from the dimer interface, we identified a greater number of steric clashes that involved R54 of one subunit and the proximal residues of the other subunit. For example, R54 of each subunit showed steric clashes with Ala-152 (A152) and Glu-153 (Q153) of the other subunit (Fig. 4B). These intra- and inter-subunit steric clashes resulted in destabilization of the T54R mutation-harboring region.

We analyzed the entropy of individual residues of the dimers of SOD1^T54R^ and SOD1 at different time points of simulation. While some of the residues of R54-loop of SOD1^T54R^ showed higher entropy during the later stages of simulation, we did not find the entropy changes of R54 and its nearby residues to be significant (Fig. 4C). Entropy values of the residues of SOD1 did not change throughout the simulation time (Fig. 4C). Entropic variation of the residues of T54-loop, including T54, was negligible.

**Fig. 5:**
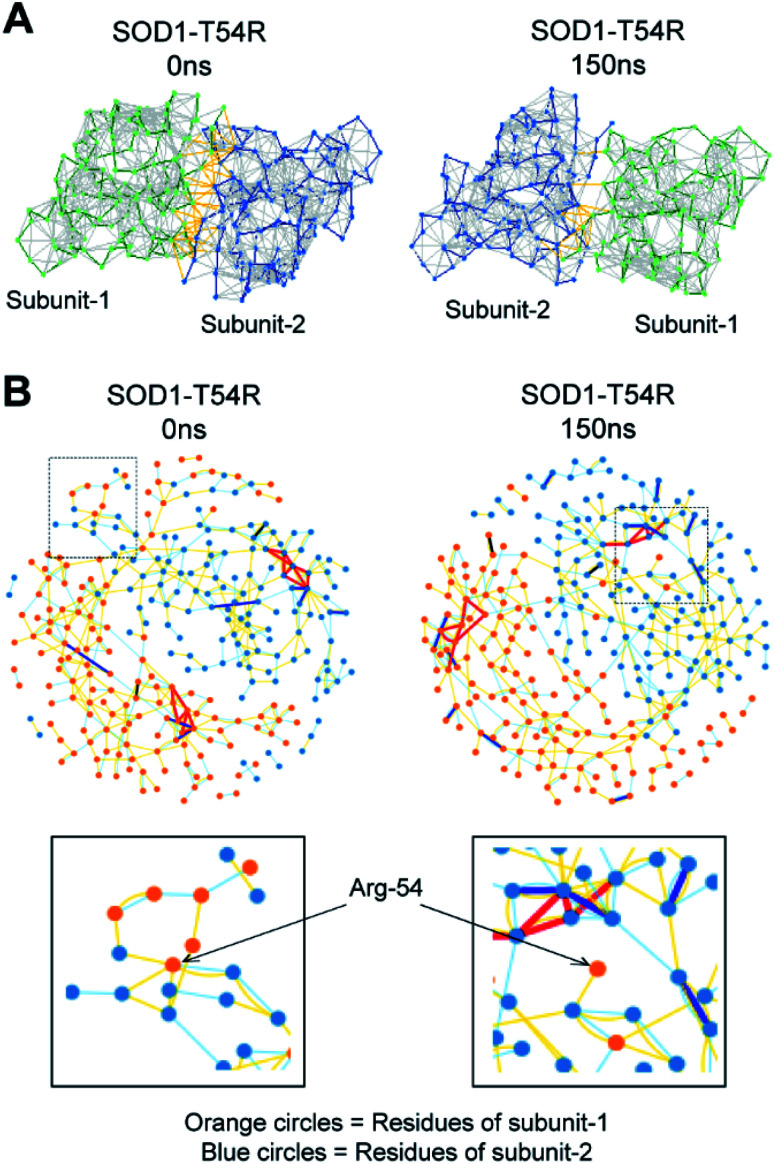
The placement of R54-loop distant from SOD1^T54R^ dimer interface results in loss of R54 interactions with other residues. (A) Noncovalent bonds at the dimers interface of SOD1^T54R^ during different time points of MDS. (B) Interaction of R54 with other residues during 0 ns and 150 ns time points of MDS.

The fluctuation of R54-loop had destroyed many stabilizing interactions at the dimer interface of SOD1^T54R^. The pre-simulation structure of SOD1^T54R^ showed abundant number of inter-subunit interactions (Fig. 5A). These interactions were comprised of hydrogen bonds, salt bridges and hydrophobic interactions. R54 of each subunit was also involved in interactions with the residues of other subunit (Fig. 5B). With the advancement of simulation time, many of the R54-mediated interactions were lost (Fig. 5A and B). Other interactions of the interface residues of SOD1^T54R^ dimer also attenuated during the simulation. These observations led us to infer that steric clashes and loss of inter-subunit interactions at the dimer interface were accounted for the instability of the SOD1^T54R^ dimer.

### Fluctuation of R54-loop has no effect on the copper and zinc binding residues of SOD1^T54R^

**Fig. 6:**
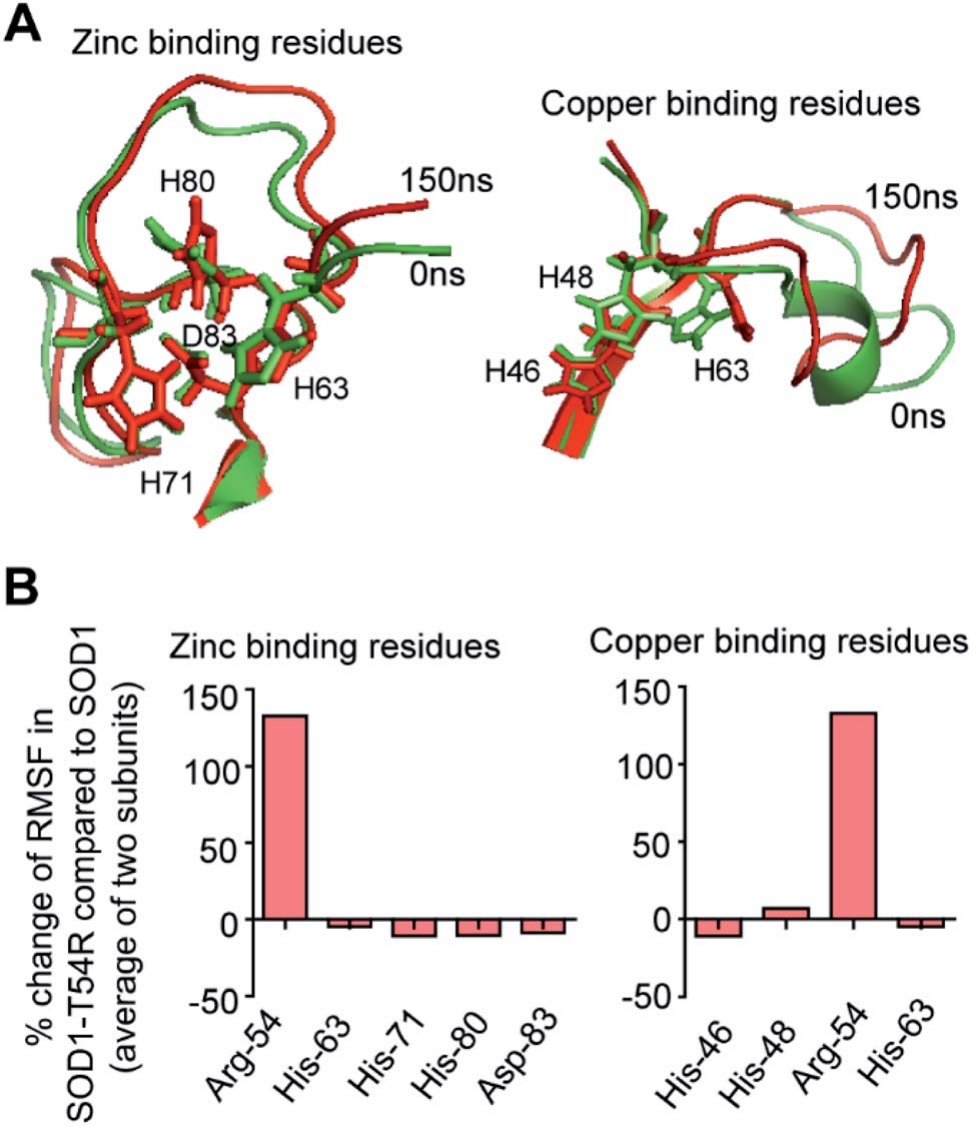
Fluctuation of R54-loop does not perturb the relative positions of zinc and copper binding residues of SOD1^T54R^. (A) Superimposed 0 ns and 150 ns positions of zinc and copper binding residues of SOD1^T54R^. (B) The percentage change of RMSF values of zinc and copper binding residues of SOD1^T54R^ in respect to RMSF values of zinc and copper binding residues of SOD1.

SOD1 contains different functional residues/regions: copper binding residues (His-46, His-48 and His-63), zinc binding residues (His-63, His-71, His-80 and Asp-83) and electrostatic loop. Since copper and zinc binding residues are catalytically important, their proper positioning in SOD1 structure is essential to coordinate the copper and zinc ions. In SOD1, the copper and zinc binding residues are separated by the T54-loop. Hence, it was necessary to understand if the fluctuation of R54-loop of SOD1^T54R^ could change the relative positions of copper and zinc binding residues, thereby affecting the catalytic efficiency of SOD1^T54R^ protein. Though R54-loop showed a high fluctuation, the zinc and copper binding residues of SOD1^T54R^ did not show high fluctuation (Fig. 6A). In fact, the RMSF values of zinc and copper binding residues of SOD1^T54R^ were less than the RMSF values of the zinc and copper binding residues of SOD1 (Fig. 6B). Zinc and copper binding residues of SOD1^T54R^ were positioned in the beta backbone that had positive phi torsion angles. Throughout the simulation time, the positive phi torsion angle was preserved in the beta-sheet structures. Since R54 was in a disordered region of SOD1^T54R^, the time-dependent fluctuation of R54-loop did not induce higher number of steric clashes by the residues of adjacent beta-sheets. Therefore, the zinc and copper binding residues did not tend to transit to negative phi torsion angle. We did not observe the collapse of beta-sheets that contained the zinc and copper binding residues, thereby maintaining the rigidity of beta-sheet structures and less fluctuation of cation binding residues.

**Fig. 7:**
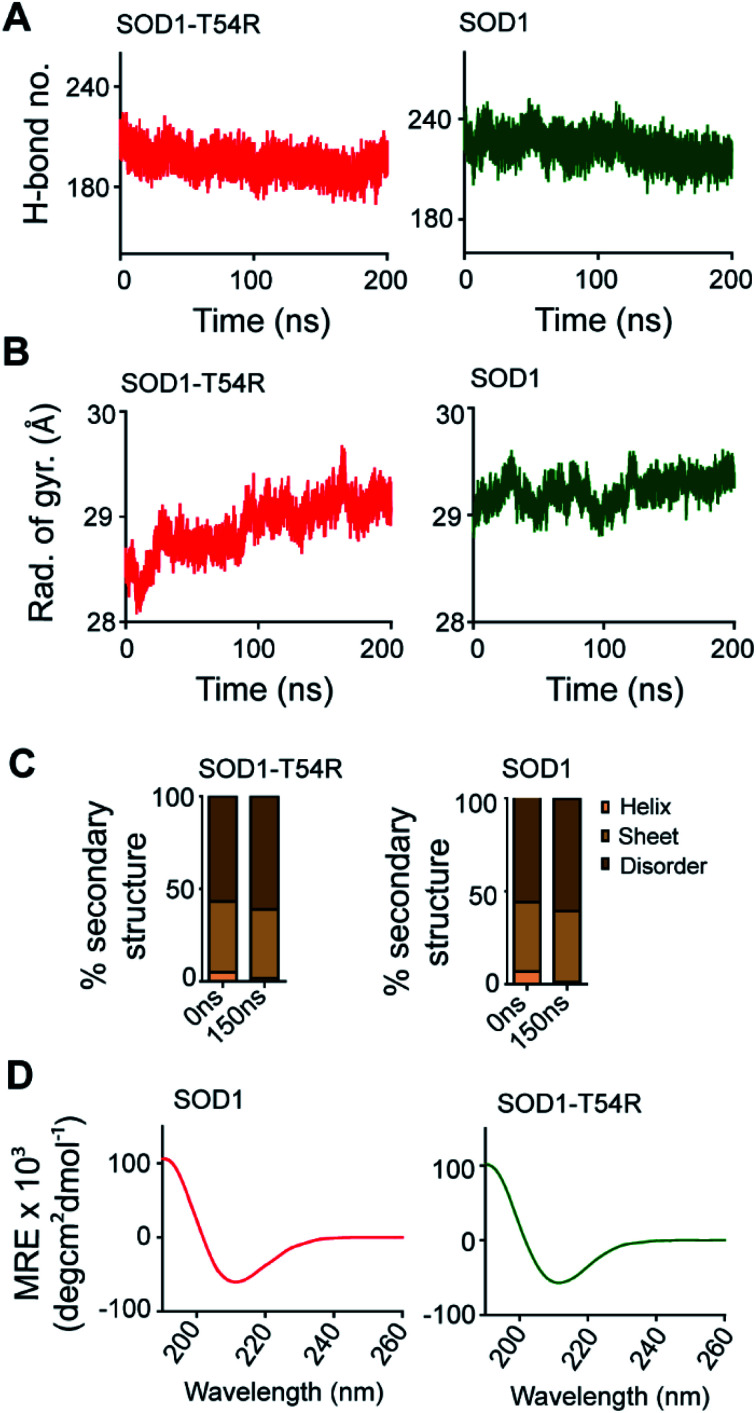
Molecular properties of SOD1^T54R^ change during progression of simulation. Simulation-associated changes of secondary structures of SOD1^T54R^ is equivalent to changes of secondary structures of SOD1. (A) The cumulative number of hydrogen bonds displayed by SOD1 and SOD1^T54R^ dimers during MDS. (B) The radius of gyration of SOD1^T54R^ and SOD1 dimers during the simulation time. (C) Secondary structure components of SOD1^T54R^ and SOD1 during different time points of simulation. (D) Circular dichroism spectroscopy of recombinant SOD1^T54R^ and SOD1 proteins.

Fluctuation of R54-loop impacted the overall shape of SOD1^T54R^ dimer. As we mentioned in an earlier section, the inter-subunit interactions, including H-bonds, of SOD1^T54R^ dimer were gradually lost during the simulation. Not only the inter-subunit H-bonds, but the cumulative number of intra-subunit H-bonds and the H-bonds between protein subunits and water were also reduced during MDS (Fig. 7A). The total number of H-bonds displayed by SOD1 dimer was higher than that of the total number of H-bonds of SOD1^T54R^ dimer (Fig. 7A). Though the total number of H-bonds manifested by SOD1 also declined over the simulation time, the number of H-bonds were still relatively higher than that of SOD1^T54R^ (Fig. 7A). The diameter of dimeric SOD1^T54R^ protein gradually increased with time. This was represented by increase of the radius of gyration of SOD1^T54R^ dimer (Fig. 7B). On the contrary, the radius of gyration of dimeric SOD1 did not show drastic changes during simulation time (Fig. 7B).

Local structural transitions are observed in the mutant SOD1 proteins. While we had earlier reported that structural transitions could occur at the edge strands of mutation-residing beta-sheets of SOD1^G85R^ and SOD1^G93A^,^21^ another study reported that trifluoroethanol could induce local unfolding of helical regions of SOD1 structure.^32^ The MDS showed that a fraction of secondary structure of SOD1^T54R^ changed during the simulation time (Fig. 7C). While beta-sheets of SOD1^T54R^ were temporally conserved, a short patch of alpha helix of R54-loop was converted to disordered structure (Fig. 7C). However, the conversion of helix-to-disorder was not exclusive for SOD1^T54R^. We observed that the wild-type SOD1 protein also showed similar loss of helical structure of the T54-loop (Fig. 7C). This was represented by decreased alpha helices and increased disordered regions in SOD1^T54R^ and SOD1 (Fig. 7C). Thus, the simulation studies showed that SOD1^T54R^ and SOD1 had nearly equal proportion of different secondary structures. We had experimentally validated this phenomenon by elucidating the secondary structures of SOD1^T54R^ and SOD1 through circular dichroism (CD) spectroscopy. The CD spectra of SOD1 and SOD1^T54R^ showed nearly identical pattern that represented abundance of beta-sheets in the structures of both proteins (Fig. 7D). So, it was evident that T54R mutation had no significant effects in altering the secondary structure composition of SOD1^T54R^ compared to SOD1.

### Exposure of R54-loop to aqueous environment transforms SOD1^T54R^ into an aggregation-prone structure

**Fig. 8:**
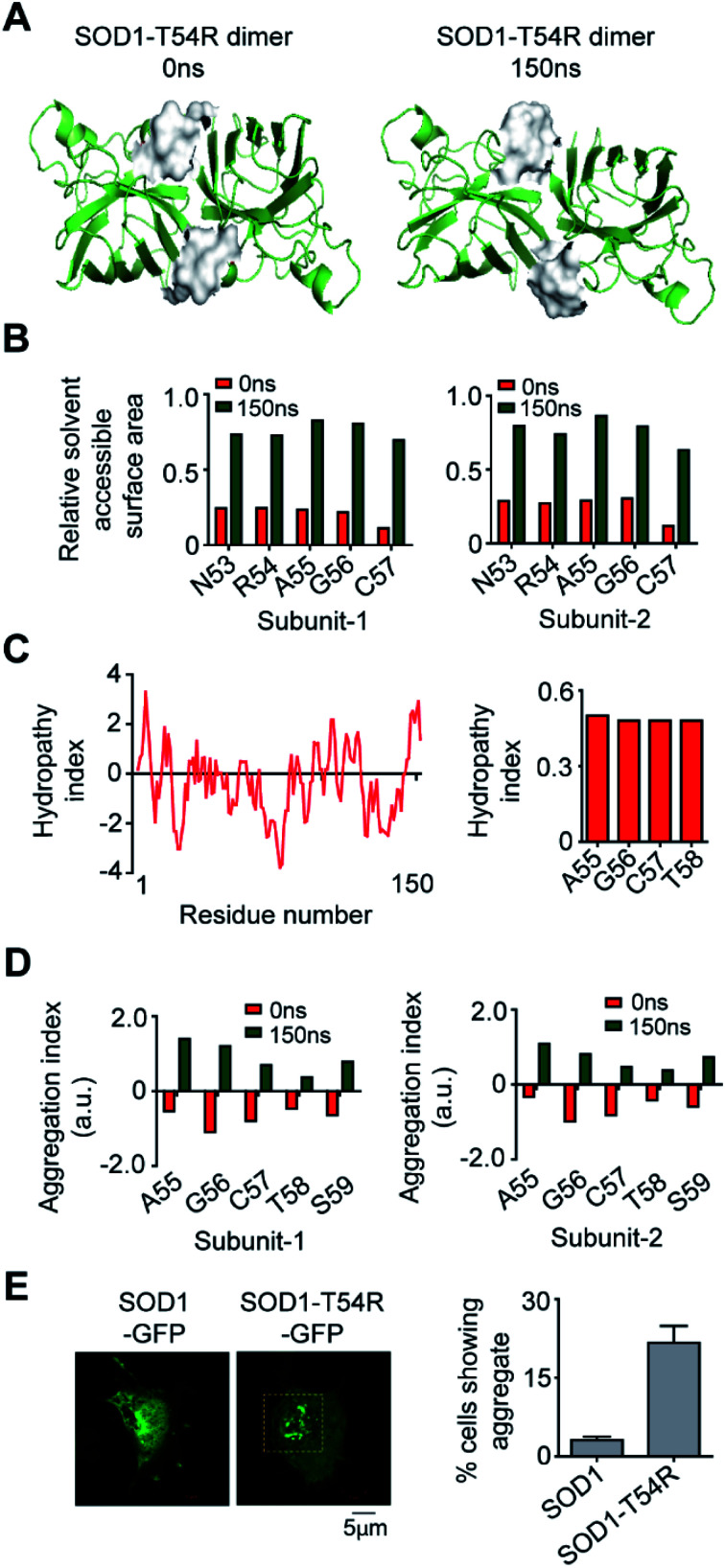
Exposure of the R54-loop towards aqueous environment make SOD1^T54R^ aggregation-prone. (A) Position of the surface of R54-loop of SOD1^T54R^ during different time points of simulation. (B) Solvent [water] accessibility of the residues of R54-loop of SOD1^T54R^ in the early and late phases of MDS. (C) Hydrophobicity of the residues of SOD1^T54^. (D) Aggregation propensities of the residues of R54-loop during different time points of MDS. (E) Fluorescence microscopy image of expression and aggregation of SOD1^T54R^-GFP in IMR-32 cells [yellow box represents aggregates of SOD1^T54R^].

In the final section, we studied if the T54R mutation had any effects on inducing aggregation properties of SOD1^T54R^. In the MDS, we observed that fluctuation of R54-loop of SOD1^T54R^ had shifted this loop to a position that was more exposed to aqueous environment (Fig. 8A). R54-loop was buried in the dimer interface cavity during the initial period of simulation. At this point, R54-loop was not exposed to aqueous environment. In the later stages of simulation, R54-loop fluctuated to an extent that resulted in the positioning of this loop distant from the interface and exposed to water. R54 and its nearby residues were prominently accessible to water in the final stages of simulation (Fig. 8B). It was noteworthy that R54-loop contained some hydrophobic residues. Though the R54-loop was amphipathic in nature, existence of the hydrophobic residues could ensue region specific (local) hydrophobicity in the loop. The hydropathy plot of SOD1^T54R^ sequence showed that the downstream residues of R54 had higher hydrophobicity index (Fig. 8C). The exposure of the hydrophobic and other residues of R54-loop to aqueous environment had increased the aggregation propensity of R54-loop of SOD1^T54R^ (Fig. 8D). Since there was no previously available report to know if SOD1^T54R^ was aggregation-prone, we conducted confocal fluorescence microscopy studies to find the expression and self-aggregation properties of SOD1^T54R^ and SOD1 in human neuroblastoma (IMR-32) cells. Both proteins were in-frame fused to green fluorescent protein (GFP) that facilitated their detection inside the cells. While we found that SOD1-GFP formed aggregates in a very few numbers of cells, SOD1^T54R^-GFP formed aggregates in higher number of cells (Fig. 8E). However, we observed that SOD1^T54R^ was not as aggregation-prone as the other mutants of SOD1 (such as SOD1^G85R^ and SOD1^G93A^). The percentage of cells showing aggregates of SOD1^T54R^ was much lower than that of the percentage of cells showing aggregates of SOD1^G93A^ (data not shown).

## Discussion

Formation of the higher order structures of proteins is a highly regulated process. Different structural signatures mediate inter-subunit association of monomers during the formation of protein oligomers. Thermodynamic and kinetic factors determine the stoichiometry of subunit numbers in multimeric protein complexes. On the other hand, uncontrolled oligomerization of aggregation-prone proteins is mediated by stochastic events. Such events result from the mutations and aberrant post-translational modifications of proteins, as well as fluctuation of environmental conditions. Very often, these changes cause misfolding of the proteins. Misfolded proteins serve as the object of uncontrolled oligomerization. Among many of the aggregation-prone proteins that are responsible for different neurodegenerative diseases, anomalous aggregation of mutant SOD1 protein leads to ALS. Given the fact that the stable SOD1 is a dimeric protein and dimer-to-monomer transition precedes the aggregation of mutant SOD1 proteins, it is a relevant question to ask how certain mutations destabilize the dimer of SOD1 protein. Our study reveals that the T54R mutation has destructive effects upon SOD1^T54R^ dimer stability due to increased fluctuation of mutation-residing loop distant from the dimer interface. Dimer-to-monomer transition makes SOD1^T54R^ more vulnerable in terms of forming aggregates.

T54 residue is situated in a very important loop region, and it is directly involved in the dimerization process of SOD1. Other than the E49-T54 loop region, several other residues of SOD1, such as V5, V7, K9, I17, I113-R115, V148-Q153, also participate in the intra-subunit contacts at dimer interface of SOD1. While most of the dimer interface residues are localized in rigid beta-sheets of SOD1, E49-T54 and I151-Q153 regions are parts of two flexible loops. Interestingly, the E49-T54 loop of each subunit interacts with the I151-Q153 loop of the other subunit of SOD1. While structural flexibility of both loops is essential for maintaining the strong holding of monomers at dimer interface, aberrant fluctuation of any of the loops can be detrimental towards the stability of SOD1 dimer. T54R mutation in SOD1^T54R^ not only changes the polar threonine residue to a positively charged arginine residue, but it also introduces a longer side chain at the mutation position. The positively charged R54 forms a transient salt-bridge with the adjacent D52 of same subunit, finally leading to a steric clash between these two residues. R54 of each subunit shows more steric clashes with Q153 of the other subunit. To lower the resultant strain of these steric clashes, the R54-loop moves distant from the dimer interface. Repositioning of E49-R54-loop causes loss of several of the intra- and inter-subunit interactions that are initially manifested by SOD1^T54R^ at it dimer interface. This leads to weakening of the interaction strength at dimer interface. Thus, the T54R mutation has unfavorable effects upon the stability of the SOD1^T54R^ dimer.

The T54 is necessary but not sufficient to cause the dimerization of SOD1. Though a high proportion of SOD1^T54R^ molecules exist as monomer in solution, a population of the protein still forms dimer. The dimerization promoting residues other than R54 of SOD^T54R^ are assumed to help the monomers to remain tethered in dimer state. However, it is also possible that the equilibrium of SOD^T54R^ shifts slowly and gradually from the dimeric state to monomeric state in a time-dependent manner.

Dimers of SOD1 are catalytically active, implying that SOD1 dimer can catalyze the conversion of superoxide ion to molecular oxygen. Previous studies have shown that some mutants of SOD1, like SOD1^G85R^, SOD1^R143D^, are catalytically inactive.^33^ In a previous MDS study of SOD1^G85R^, we have also shown that deviation of electrostatic loop of SOD1^G85R^ destroys the substrate guiding channel, thereby rendering the protein catalytically inactive, although the copper and zinc binding residues are potentially able to bind copper and zinc ions.^21^ In this study, we find that T54R mutation destabilizes the dimer of SOD1^T54R^ without possibly altering the catalytic activity of the protein. Even though R54-loop of SOD1^T54R^ fluctuates to disrupt the dimer interface, it does not perturb the structural segments that contain the copper and zinc ion binding residues. Moreover, the intrinsic fluctuation of the electrostatic loop was also reduced in SOD1^T54R^ than that of wild-type SOD1. Thus, SOD1^T54R^ can be even more conducive to efficient catalysis. However, this phenomenon remains to be experimentally proven.

Transition of dimers to monomers is fundamentally associated with higher aggregation of mutant SOD1 proteins. For example, destabilized monomers of SOD1^G37R^, SOD1^G93A^ and SOD1^V148G^ show higher aggregation. We find that it is also true for SOD1^T54R^. T54R mutation decreases the stability of dimer interface which subsequently increases the quantitative pool of monomeric SOD1^T54R^. Such higher numbers of monomeric SOD1^T54R^ can form intracellular aggregates. However, we note that SOD1^T54R^ protein's capacity of forming aggregates is less than the aggregation potential of SOD1^G85R^ and SOD1^G93A^. The aggregation properties of SOD1^T54R^ arise from the increased exposure of the hydrophobic residues of R54-loop to the aqueous solvent during the reorientation of R54-loop and dimer-to-monomer transition of SOD1^T54R^.

Overall, our study provides a comprehensive understanding of how the T54R mutation induces steric clashes at the dimer interface that eventually reduces the stability of dimers of SOD1^T54R^. The study also illustrates the mechanism of aggregation of SOD1^T54R^ that possibly does not interfere in the catalytic capacity of the protein.

## Methods

### Structures

The structures of apo-SOD1 and apo-SOD1^T54R^ were curated from the protein data bank. The PDB codes 3ECU^34^ and 3ECW^34^ represented the SOD1 and SOD1^T54R^ structures respectively. The dimeric structures of SOD1 and SOD1^T54R^ were prepared from the original structures of the proteins.

### Molecular dynamics simulation

The conformational dynamics of the dimers of SOD1 and SOD1^T54R^ were analyzed by molecular dynamics simulation (MDS). The simulations were performed in the same process that was done by us in our earlier studies.^21,35,36^ There were three sequential steps in the MDS process – (a) protein structure preparation: in this step, optimization of protein structure was done to add missing hydrogen atoms, generate missing inter-atomic bonds and assign correct bond orders. Protein structure preparation was done in the protein preparation wizard of Maestro 9.2 (Schrödinger Incorporation) by using the following parameters – OPLS_2005 force field, 0.3 Å convergence heavy atom root mean square deviation. Protein structure was minimized in the molecular modelling tool kit (MMTK)^37^ with amber parameters.^38^ This involved 10^3^ iterations of steepest descent in conjugate gradient minimization. (b) Simulation environment generation: the simulation environment was generated by solvating protein structure in virtual water molecules by using TIP3P^39^ model in the system builder of Desmond (Schrödinger Incorporation). Water molecules were placed in a cuboidal geometry up to 40 Å from the protein molecule. 150 mM Na^+^ and Cl^−^ were added in water environment to neutralize the charged residues of protein. Periodic boundary condition was applied in every dimension. In the solvated medium, protein structure was minimized by using following parameters – 2000 iteration of 1 kcal mole^−1^ Å^−1^ convergence threshold steepest descent minimization. High-energy contacts in protein molecule were eliminated in the minimization process by using the OPLS_2005 force field. (c) Molecular dynamics simulation – the molecular dynamics simulation of protein was done in Desmond of Maestro 9.2 (Schrödinger Incorporation). The MDS process comprised two sequential steps – (c-I) equilibrium simulation: an equilibrium simulation of protein structure for 2 ns was done to allow complete relaxation of the structures and the water environment. The equilibrium simulation had discrete steps which were as follows – solute's restrained minimization of solutes, unrestrained minimization, constant particle number–volume–temperature (NVT) simulation with restraints on the heavy atoms of solutes, constant particle number–pressure–temperature (NPT) simulation with restraints on particles heavy atoms and an unrestrained simulation. (c-II) Unrestrained full simulation: the final step in MDS was the unrestrained 200 ns simulation with following simulation conditions – NPT ensemble class, 298 K temperature and the Nose–Hoover chain thermostat method^40^ to maintain the constant temperature with 1 ps relaxation time, maintenance of constant pressure of 1.01325 bar by Martyna–Tobias–Klein barostat method^41^ with isotropic coupling and 2 ps relaxation time, RESPA integrator (6 fs far, 2 fs near and 2 fs bonded),^42,43^ randomized velocity coupled OPLS_2005 force field, 1 nm cutoff short range interactions, long range interactions (smooth particle mesh Ewald long-range coulombic interaction with the Ewald^44^ tolerance of 10^−9^). The recording of frames of trajectories were done at an interval of 200 ps.

Simulation interaction diagram generator of Maestro 9.2 (Schrödinger Incorporation) was used to analyze the temporal pattern of root mean square deviation (RMSD) of protein backbone and the root mean square fluctuation (RMSF) of the protein residues.

Simulation event analysis program of Maestro 9.2 (Schrödinger Incorporation) was used to calculate the time dependent changes of radius of gyration of protein and the number of hydrogen bonds manifested by protein.

The accessible surface area and the free energy change of protein structure at different time points of MDS were also calculated in Desmond of Maestro 9.2 (Schrödinger Incorporation).

### Protein–protein interaction analysis

Analysis of the inter-subunit interactions of SOD1 and SOD1^T54R^ were done in the PDBePISA (Proteins, Interfaces, Structures and Assemblies) Web server.^45^ The algorithm of PDBePISA measured several properties, like buried surface area at interface, interacting residues at interface, Δ*G*^int^ (gain of solvation free energy upon formation of dimer), Δ*G*^diss^ (free energy of dimer disassembly; Δ*G*^diss^ > 0 is a thermodynamically stable dimer), number of hydrogen bonds at interface, entropy of assembly (*T*Δ*S*) *etc.*, of the subunit interfaces of SOD1 and SOD1^T54R^ dimer. Analysis were done for the interfaces of SOD1 and SOD1^T54R^ dimers at different time points of MDS.

### Steric clash analysis

Steric clashes between the intra- or inter-subunit residues were analyzed in Molprobity Web server.^46^

### Conformational entropy analysis of protein residues

The conformational entropy of the residues of dimeric SOD1 and SOD1^T54R^ at different time points of MDS were analyzed in PLOPS Web server.^47^

### Construction of residue interaction network and analysis of inter-residue interactions

The interaction networks of different residues of dimeric SOD1 and SOD1^T54R^ were generated in the RING2.0 (Residue Interaction Network Generator) Web server.^48^ This visualization tool pictorially demonstrated all the noncovalent bonds that existed between the residues of dimeric SOD1 and SOD1^T54R^. Interaction networks and structure contacts were generated in Cytoscape^49^ and Pymol^50^ respectively.

### Hydropathy index analysis

The hydrophobicity of different regions of SOD1 and SOD1^T54R^ were analyzed in the ProtScale ExPASy Web server (https://web.expasy.org/protscale/).

### Aggregation property analysis of protein residues

The aggregation index of different residues of SOD1 and SOD1^T54R^ at different time points of MDS was analyzed in Aggrescan3D Web server.^51^

### Cloning

Following the total mRNA isolation from IMR-32 cells, cDNA of SOD1 was synthesized by using oligo-dT primer in RT-PCR. The dsDNA corresponding to SOD1 ORF was synthesized by PCR using SOD1 specific forward and reverse primers. T54R mutation was introduced in the wild-type sequence of SOD1 by site-directed mutagenesis which was done by overlapping PCR based method using two pairs of primer sets. PCR products of SOD1 and SOD1^T54R^ were separately inserted in pET21b and pEGFP-N1 vectors by using conventional cloning procedure. The overall cloning process was similar to what was described in our earlier studies.^52–54^ PCR products and plasmids were digested by specific restriction enzymes, followed by ligation of PCR products with the corresponding plasmids. Ligated products were transformed into the ultra-competent DH5α strain of *Escherichia coli*. Viable colonies were selected on the specific antibiotic-containing LB-agar plate and positive clones were identified by colony-PCR. All positive clones were sequences at the sophisticated equipment facility of research support service group of CDFD.

### Recombinant protein production and purification

The recombinant SOD1 and SOD1^T54R^ proteins were expressed in the BL21DE3 strain of *Escherichia coli* using T7 expression system. Briefly, the bacterial expression clones of SOD1 and SOD1^T54R^ (SOD1-pET21b and SOD1^T54R^-pET21b) were separately transformed in the BL21DE3 strain of *Escherichia coli*, followed by induction of protein production by application of 1 mM IPTG in the culture medium. 16 hours (at 37 °C) after IPTG treatment, bacterial cells were harvested and lysed in lysis buffer [50 mM NaH_2_PO_4_ (pH: 8.0), 300 mM NaCl, 10 mM imidazole and 1 mM PMSF]. Cleared lysate was passed through Ni-NTA column to allow the binding of 6xHistidine containing recombinant proteins to the column. This was followed by washing of the protein-bound column with wash buffer [50 mM NaH_2_PO_4_ (pH: 8.0), 300 mM NaCl, 40 mM imidazole] and elution of protein in elution buffer [50 mM NaH_2_PO_4_ (pH: 8.0), 300 mM NaCl, 300 mM imidazole]. Proteins were dialyzed in dialysis buffer [20 mM NaH_2_PO_4_ (pH: 7.5), 20 mM NaCl]. In general, the process of recombinant protein expression and purification was similar to method that was described in our earlier studies.^55,56^

### Immunoblotting

Immunoblotting of recombinant SOD1 and SOD1^T54R^ proteins was done to analyze the oligomerization status of SOD1 and SOD1^T54R^. 40 μg of SOD1 and SOD1^T54R^ proteins were separated in a native (nondenaturing) PAGE, followed by transfer of proteins from the gel to PVDF membrane. The membrane was sequentially treated with monoclonal anti-polyHistidine primary antibody (Sigma-Aldrich, H1029, dilution – 1:5000) and anti-mouse IgG (whole molecule)–peroxidase secondary antibody (Sigma-Aldrich, A9044, dilution – 1:5000). Intermittent blocking and washing of the membrane were done by 5% skim-milk (in TBS buffer, pH: 7.4) and TBST (pH: 7.5) buffer.

### Circular dichroism spectroscopy

Circular dichroism (CD) spectroscopy of recombinant SOD1 and SOD1^T54R^ proteins was done to analyze the secondary structure pattern of these proteins. Proteins were kept in 50 mM NaH_2_PO_4_ (pH: 7.4), 50 mM NaF buffer. CD spectra of proteins were taken in the far ultraviolet wavelength range (190–260 nm) at 25 °C. Spectroscopic measurements were done by keeping protein in a 10 mm path length cuvette of JASCO 810 spectropolarimeter. The method of CD spectroscopy was similar to the process that was described in one of our earlier studies.^36^

### Cell culture

IMR-32 cells were obtained from National Centre for Cell Science (India). Cells were cultured in Dulbecco's Modified Eagle Medium that was supplemented with 10% fetal bovine serum, 2 mM l-glutamine and penicillin/streptomycin solution. Cultured cells were maintained at 37 °C in a humified incubator.

Transfection of SOD1 and SOD1^T54R^ clones (SOD1-pEGFP-N1 and SOD1^T54R^-pEGFP-N1) into IMR-32 cells were done by using Lipofectamine-2000 (Thermo Fisher Scientific) reagent.

### Fluorescence microscopy

Monitoring the expression and aggregation of SOD1-EGFP and SOD1^T54R^-EGFP in IMR-32 cells were done by fluorescence microscopy. Cells were fixed by 4% paraformaldehyde (in PBS, pH: 7.4), followed by blocking of the cells with 1% bovine serum albumin (in PBS, pH: 7.4) and intermittent washing with PBS (pH: 7.4). Image acquisition was done in LSM700 (Zeiss) confocal laser scanning microscope using 63× Plan Apo/1.4 NA oil immersion objective. Image processing was done in Zen-lite (Zeiss) software. The overall process of cell preparation and microscopy was similar to one of our previous studies.^57^

## Data availability

All data related to this study are available on request from the authors.

## Author contributions

AR and DKG made the hypothesis and objectives of the study. DKG performed all the studies. AK contributed in cloning and CD spectroscopy. AR supervised the study and analyzed the results. AR and DKG wrote the manuscript.

## Conflicts of interest

The authors have no potential conflict of interests to disclose.

## Supplementary Material

## References

[cit1] Robertson A. D., Murphy K. P. (1997). Protein Structure and the Energetics of Protein Stability. Chem. Rev..

[cit2] Teilum K., Olsen J. G., Kragelund B. B. (2011). Protein stability, flexibility and function. Biochim. Biophys. Acta.

[cit3] Fontana A. (1991). Analysis and modulation of protein stability. Curr. Opin. Biotechnol..

[cit4] Kim Y. E., Hipp M. S., Bracher A., Hayer-Hartl M., Hartl F. U. (2013). Molecular chaperone functions in protein folding and proteostasis. Annu. Rev. Biochem..

[cit5] Knorre D. G., Kudryashova N. V., Godovikova T. S. (2009). Chemical and functional aspects of posttranslational modification of proteins. Acta Naturae.

[cit6] Brinda K. V., Vishveshwara S. (2005). Oligomeric protein structure networks: insights into protein-protein interactions. BMC Bioinf..

[cit7] Chi E. Y., Krishnan S., Randolph T. W., Carpenter J. F. (2003). Physical stability of proteins in aqueous solution: mechanism and driving forces in nonnative protein aggregation. Pharm. Res..

[cit8] Zhang Z., Miteva M. A., Wang L., Alexov E. (2012). Analyzing effects of naturally occurring missense mutations. Computational and Mathematical Methods in Medicine.

[cit9] Ashenberg O., Gong L. I., Bloom J. D. (2013). Mutational effects on stability are largely conserved during protein evolution. Proc. Natl. Acad. Sci. U. S. A..

[cit10] Jones S., Marin A., Thornton J. M. (2000). Protein domain interfaces: characterization and comparison with oligomeric protein interfaces. Protein Eng..

[cit11] Pace C. N., Shirley B. A., McNutt M., Gajiwala K. (1996). Forces contributing to the conformational stability of proteins. FASEB J..

[cit12] Frieden C. (2007). Protein aggregation processes: in search of the mechanism. Protein Sci..

[cit13] Jamadagni S. N., Godawat R., Garde S. (2011). Hydrophobicity of proteins and interfaces: insights from density fluctuations. Annu. Rev. Chem. Biomol. Eng..

[cit14] Fink A. L. (1998). Protein aggregation: folding aggregates, inclusion bodies and amyloid. Folding Des..

[cit15] Bunton-Stasyshyn R. K., Saccon R. A., Fratta P., Fisher E. M. (2015). SOD1 Function and Its Implications for Amyotrophic Lateral Sclerosis Pathology: New and Renascent Themes. Neuroscientist.

[cit16] Valentine J. S., Doucette P. A., Zittin Potter S. (2005). Copper-zinc superoxide dismutase and amyotrophic lateral sclerosis. Annu. Rev. Biochem..

[cit17] Auclair J. R., Boggio K. J., Petsko G. A., Ringe D., Agar J. N. (2010). Strategies for stabilizing superoxide dismutase (SOD1), the protein destabilized in the most common form of familial amyotrophic lateral sclerosis. Proc. Natl. Acad. Sci. U. S. A..

[cit18] Banci L., Bertini I., Boca M., Girotto S., Martinelli M., Valentine J. S., Vieru M. (2008). SOD1 and amyotrophic lateral sclerosis: mutations and oligomerization. PLoS One.

[cit19] Julien J. P. (2001). Amyotrophic lateral sclerosis. Unfolding the toxicity of the misfolded. Cell.

[cit20] Taylor J. P., Brown Jr R. H., Cleveland D. W. (2016). Decoding ALS: from genes to mechanism. Nature.

[cit21] Ghosh D. K., Shrikondawar A. N., Ranjan A. (2020). Local structural unfolding at the
edge-strands of beta-sheets is the molecular basis for instability and aggregation of G85R and G93A mutants of Superoxide dismutase 1. J. Biomol. Struct. Dyn..

[cit22] Munch C., Bertolotti A. (2010). Exposure of hydrophobic surfaces initiates aggregation of diverse ALS-causing superoxide dismutase-1 mutants. J. Mol. Biol..

[cit23] Lee S., Kim H. J. (2015). Prion-like Mechanism in Amyotrophic Lateral Sclerosis: are Protein Aggregates the Key?. Exp. Neurol..

[cit24] McAlary L., Aquilina J. A., Yerbury J. J. (2016). Susceptibility of Mutant SOD1 to Form a Destabilized Monomer Predicts Cellular Aggregation and Toxicity but Not In vitro Aggregation Propensity. Front. Neurosci..

[cit25] Ding F., Furukawa Y., Nukina N., Dokholyan N. V. (2012). Local unfolding of Cu, Zn superoxide dismutase monomer determines the morphology of fibrillar aggregates. J. Mol. Biol..

[cit26] Broom H. R., Rumfeldt J. A., Vassall K. A., Meiering E. M. (2015). Destabilization of the dimer interface is a common consequence of diverse ALS-associated mutations in metal free SOD1. Protein Sci..

[cit27] Karch C. M., Prudencio M., Winkler D. D., Hart P. J., Borchelt D. R. (2009). Role of mutant SOD1 disulfide oxidation and aggregation in the pathogenesis of familial ALS. Proc. Natl. Acad. Sci. U. S. A..

[cit28] Ray S. S., Nowak R. J., Strokovich K., Brown Jr R. H., Walz T., Lansbury Jr P. T. (2004). An intersubunit disulfide bond prevents in vitro aggregation of a superoxide dismutase-1 mutant linked to familial amyotrophic lateral sclerosis. Biochemistry.

[cit29] Andersen P. M., Sims K. B., Xin W. W., Kiely R., O'Neill G., Ravits J., Pioro E., Harati Y., Brower R. D., Levine J. S., Heinicke H. U., Seltzer W., Boss M., Brown Jr R. H. (2003). Sixteen novel mutations in the Cu/Zn superoxide dismutase gene in amyotrophic lateral sclerosis: a decade of discoveries, defects and disputes. Amyotrophic Lateral Scler. Other Mot. Neuron Disord..

[cit30] Xu Y., Li S., Yan Z., Ge B., Huang F., Yue T. (2019). Revealing Cooperation between Knotted Conformation and Dimerization in Protein Stabilization by Molecular Dynamics Simulations. J. Phys. Chem. Lett..

[cit31] Xu Y., Li S., Yan Z., Luo Z., Ren H., Ge B., Huang F., Yue T. (2018). Stabilizing Effect of Inherent Knots on Proteins Revealed by Molecular Dynamics Simulations. Biophys. J..

[cit32] Kumar V., Prakash A., Pandey P., Lynn A. M., Hassan M. I. (2018). TFE-induced local unfolding and fibrillation of SOD1: bridging the experiment and simulation studies. Biochem. J..

[cit33] Liu J., Lillo C., Jonsson P. A., Vande Velde C., Ward C. M., Miller T. M., Subramaniam J. R., Rothstein J. D., Marklund S., Andersen P. M., Brannstrom T., Gredal O., Wong P. C., Williams D. S., Cleveland D. W. (2004). Toxicity of familial ALS-linked SOD1 mutants from selective recruitment to spinal mitochondria. Neuron.

[cit34] Banci L., Bertini I., Boca M., Calderone V., Cantini F., Girotto S., Vieru M. (2009). Structural and dynamic aspects related to oligomerization of apo SOD1 and its mutants. Proc. Natl. Acad. Sci. U. S. A..

[cit35] Ghosh D. K., Kumar A., Ranjan A. (2018). Metastable states of HYPK-UBA domain's seeds drive the dynamics of its own aggregation. Biochim.
Biophys. Acta, Gen. Subj..

[cit36] Ghosh D. K., Roy A., Ranjan A. (2018). Disordered Nanostructure in Huntingtin Interacting Protein K Acts as a Stabilizing Switch To Prevent Protein Aggregation. Biochemistry.

[cit37] Hinsen K. (2000). The Molecular Modeling Toolkit: A New Approach to Molecular Simulations. J. Comput. Chem..

[cit38] Case D. A., Cheatham T. E., Darden T., Gohlke H., Luo R., Merz Jr K. M., Onufriev A., Simmerling C., Wang B., Woods R. J. (2005). The Amber biomolecular simulation programs. J. Comput. Chem..

[cit39] Baranyai A., Evans D. J. (1989). Direct entropy calculation from computer simulation of liquids. Phys. Rev. A: At., Mol., Opt. Phys..

[cit40] Evans D. J., Holian B. L. (1985). The Nose–Hoover thermostat. J. Chem. Phys..

[cit41] Martyna G. J., Tobias D. J., Klein M. L. (1994). Constant pressure molecular dynamics algorithms. J. Chem. Phys..

[cit42] Tuckerman M., Berne B. J. (1991). Molecular dynamics algorithm for multiple time scales: systems with disparate masses. J. Chem. Phys..

[cit43] Tuckerman M., Berne B. J. (1992). Reversible multiple time scale molecular dynamics. J. Chem. Phys..

[cit44] Ewald P. (1921). Die Berechnung optischer und elektrostatischer Gitterpotentiale. Ann. Phys..

[cit45] Krissinel E., Henrick K. (2007). Inference of macromolecular assemblies from crystalline state. J. Mol. Biol..

[cit46] Chen V. B., Arendall W. B., Headd J. J., Keedy D. A., Immormino R. M., Kapral G. J., Murray L. W., Richardson J. S., Richardson D. C. (2010). MolProbity: all-atom structure validation for macromolecular crystallography. Acta Crystallogr., Sect. D: Biol. Crystallogr..

[cit47] Baxa M. C., Haddadian E. J., Jumper J. M., Freed K. F., Sosnick T. R. (2014). Loss of conformational entropy in protein folding calculated using realistic ensembles and its implications for NMR-based calculations. Proc. Natl. Acad. Sci. U. S. A..

[cit48] Piovesan D., Minervini G., Tosatto S. C. (2016). The RING 2.0 web server for high quality residue interaction networks. Nucleic Acids Res..

[cit49] Shannon P., Markiel A., Ozier O., Baliga N. S., Wang J. T., Ramage D., Amin N., Schwikowski B., Ideker T. (2003). Cytoscape: a software environment for integrated models of biomolecular interaction networks. Genome Res..

[cit50] DeLano W. L. P. , An open-source molecular graphics tool, CCP4 Newsletter On Protein Crystallography, 2002, vol. 40, pp. 82–92

[cit51] Zambrano R., Jamroz M., Szczasiuk A., Pujols J., Kmiecik S., Ventura S. (2015). AGGRESCAN3D (A3D): server for prediction of aggregation properties of protein structures. Nucleic Acids Res..

[cit52] Ghosh D. K., Roy A., Ranjan A. (2018). Aggregation-prone Regions in HYPK Help It to Form Sequestration Complex for Toxic Protein Aggregates. J. Mol. Biol..

[cit53] Kumar A., Ghosh D. K., Ali J., Ranjan A. (2019). Characterization of Lipid Binding Properties of Plasmodium falciparum Acyl-Coenzyme A Binding Proteins and Their Competitive Inhibition by Mefloquine. ACS Chem. Biol..

[cit54] Kumar A., Ghosh D. K., Ranjan A. (2020). Mefloquine binding to human acyl-CoA
binding protein leads to redox stress-mediated apoptotic death of human neuroblastoma cells. Neurotoxicology.

[cit55] Roy A., Reddi R., Sawhney B., Ghosh D. K., Addlagatta A., Ranjan A. (2016). Expression, Functional Characterization and X-ray Analysis of HosA, A Member of MarR Family of Transcription Regulator from Uropathogenic Escherichia coli. Protein J..

[cit56] Ghosh D. K., Roy A., Ranjan A. (2018). The ATPase VCP/p97 functions as a disaggregase against toxic Huntingtin-exon1 aggregates. FEBS Lett..

[cit57] Ghosh D. K., Ranjan A. (2019). An IRES-dependent translation of HYPK mRNA generates a truncated isoform of the protein that lacks the nuclear localization and functional ability. RNA Biol..

